# Inhibitory effect of a TGF*β* receptor type-I inhibitor, Ki26894, on invasiveness of scirrhous gastric cancer cells

**DOI:** 10.1038/sj.bjc.6605561

**Published:** 2010-02-09

**Authors:** O Shinto, M Yashiro, H Kawajiri, K Shimizu, T Shimizu, A Miwa, K Hirakawa

**Affiliations:** 1 Department of Surgical Oncology, Osaka City University Graduate School of Medicine, Osaka, Japan; 2 Oncology Institute of Geriatrics and Medical Science, Osaka City University Graduate School of Medicine, Osaka, Japan; 3 Pharmacological Research Laboratories, Kyowa Hakko Kirin Co Ltd, Gunma, Japan; 4 Research Planning Department, Kyowa Hakko Kirin Co Ltd, Gunma, Japan; 5 Drug Discovery Research Laboratories, Kyowa Hakko Kirin Co Ltd, Gunma, Japan

**Keywords:** scirrhous gastric cancer, TGF-*β*, epithelial-to-mesenchymal transition, Smad2, phosphorylation inhibitor

## Abstract

**Background:**

Gastric cancer cells frequently metastasise, partly because of their highly invasive nature. Transforming growth factor-*β* (TGF-*β*) receptor signalling is closely associated with the invasion of cancer cells. The aim of this study was to clarify the effect of a TGF-*β* receptor (T*β*R) phosphorylation inhibitor on the invasiveness of gastric cancer cells.

**Methods:**

Four gastric cancer cell lines, including two scirrhous-type cell lines and two non-scirrhous-type cell lines, were used. A T*β*R type I (T*β*R-I) kinase inhibitor, Ki26894, inhibits the phosphorylation of Smad2 at an ATP-binding site of T*β*R-I. We investigated the expression levels of T*β*R and phospho-Smad2, and the effects of TGF-*β* in the presence or absence of Ki26894 on Smad2 phosphorylation, invasion, migration, epithelial-to-mesenchymal transition (EMT), Ras homologue gene family member A (RhoA), ZO-2, myosin, and E-cadherin expression of gastric cancer cells.

**Results:**

T*β*R-I, T*β*R-II, and phospho-Smad2 expressions were found in scirrhous gastric cancer cells, but not in non-scirrhous gastric cancer cells. Ki26894 decreased Smad2 phosphorylation induced by TGF-*β*1 in scirrhous gastric cancer cells. Transforming growth factor-*β*1 upregulated the invasion, migration, and EMT ability of scirrhous gastric cancer cells. Transforming growth factor-*β*1 significantly upregulated the activity of RhoA and myosin phosphorylation, whereas TGF-*β*1 decreased ZO-2 and E-cadherin expression in scirrhous gastric cancer cells. Interestingly, Ki26894 inhibited these characteristics in scirrhous gastric cancer cells. In contrast, non-scirrhous gastric cancer cells were not affected by TGF-*β*1 or Ki26894 treatment.

**Conclusion:**

A T*β*R-I kinase inhibitor decreases the invasiveness and EMT of scirrhous gastric cancer cells. Ki26894 is therefore considered to be a promising therapeutic compound for the metastasis of scirrhous gastric carcinoma.

There are two types of gastric cancer: diffuse type and intestinal type, according to the Laurén classification (Lauren, 1965). Scirrhous gastric carcinoma, also called diffuse-type gastric carcinoma, or linitis plastica, is characterised by a diffusely infiltrating growth accompanied by extensive stromal fibrosis and a high frequency of metastasis to the lymph nodes and the peritoneum (Yokota *et al*, 1999; Nakazawa *et al*, 2003), resulting in an extremely poor prognosis (Otsuji *et al*, 2004; Kunisaki *et al*, 2005). The high invasive ability of cancer cells is responsible for the diffusely infiltrating growth and frequent metastatic spread of scirrhous gastric carcinoma.

Transforming growth factor-*β* (TGF-*β*) signals have an important role in the metastatic spread of cancer cells (Saito *et al*, 2000; Massague, 2008; Yang and Moses, 2008), such as migration, invasion, and epithelial-to-mesenchymal transition (EMT) (Thiery, 2002; Massague, 2008). Overexpression of TGF-*β* is reported to be correlated with poor prognosis of gastric tumours (Saito *et al*, 2000), especially scirrhous gastric carcinoma (Kinugasa *et al*, 1998), suggesting that TGF-*β* signalling might have an important role in the progression of scirrhous gastric cancer cells (Inoue *et al*, 1997; Kinugasa *et al*, 1998; Kawajiri *et al*, 2008). Therefore, inhibition of TGF-*β* signalling in scirrhous gastric carcinoma may yield beneficial effects through inhibition of invasion and metastasis of cancer. Although there are several reports of molecular target therapies against TGF-*β* signalling from the viewpoint of adhesion (Kano *et al*, 2007; Kawajiri *et al*, 2008), proliferation (Tateishi *et al*, 2000; Komuro *et al*, 2009), or angiogenesis (Kano *et al*, 2007; Kawajiri *et al*, 2008) in scirrhous gastric carcinoma, the effectiveness of TGF-*β* signalling inhibitor on invasion and migration has not been proposed in this type of carcinoma. Transforming growth factor-*β* activates TGF-*β* receptor type II (T*β*R-II), which phosphorylates TGF-*β* receptor type I (T*β*R-I) (Heldin *et al*, 1997; Massague, 2008). Activated T*β*R-I kinase phosphorylates Smad2/3, which are associated with Smad4 and translocation in the nucleas as transcriptional factors (Heldin *et al*, 1997; Massague, 2008). In this study, we investigated the effect of a novel T*β*R-I inhibitor Ki26894 on the invasiveness of gastric cancer.

## Materials and methods

### Compounds

A small synthetic molecule that interrupts the phosphorylation of Smad2/Smad3 by T*β*R-I, namely, Ki26894, was synthesised by Kirin Brewery Company (Gunma, Japan) as previously reported (Ehata *et al*, 2007). Ki26894 was dissolved in PBS (Nikken Bio., Kyoto, Japan), stored at 4°C, and used within 5 days. Transforming growth factor-*β*1 was purchased from R&D Systems (Minneapolis, MN, USA).

### Cell lines

OCUM-2MLN (Fujihara *et al*, 1999) and OCUM-12 (Qiu *et al*, 2009) were derived from scirrhous gastric carcinomas. MKN-45 (Motoyama *et al*, 1986) and MKN-74 (Motoyama *et al*, 1986) were derived from non-scirrhous gastric carcinomas. The culture medium consisted of DMEM (Nikken Bio.) with the addition of 10% heat-inactivated fetal bovine serum (FBS; Equitech-Bio, Kerrville, TX, USA), 100 IU ml^−1^ penicillin (ICN Biomedicals, Costa Mesa, CA, USA), 100 *μ*g ml^−1^ streptomycin (ICN Biomedicals), and 0.5 mM sodium pyruvate (Cambrex, Walkersville, MD, USA). Cells were cultured at 37°C in a humidified atmosphere containing 5% CO_2_ in the air.

### Morphological findings

Cancer cells were also cultured with TGF-*β*1 (10 ng ml^−1^) and/or Ki26894 (0.1, 0.3, 1, 3, or 10 *μ*M). Cell morphology was observed microscopically, 24, 48, and 72 h after addition.

### Wound healing assay

*In vitro* wound healing ability was measured by the method described in the study by Borensztajn *et al* (2008), with some modifications. Gastric cancer cells were cultured in six-well plates. After the cells reached semi-confluence, a wound was created in the cell monolayer using a pipette tip. Cancer cells were cultured in serum-free DMEM, along with TGF-*β*1 (10 ng ml^−1^) and/or Ki26894 (10 *μ*M). Four scratched fields were randomly chosen and the number of cell migrations was counted from pictures 24 h after treatment.

### Invasion assay

*In vitro* invasiveness was measured by the method described in the study by Albini *et al* (1987), with some modifications. We used chemotaxis chambers with a 12 *μ*m-pore membrane filter (Kubota, Osaka, Japan) coated with 50 *μ*g of matrigel in a 24-well culture plate. Gastric cancer cells (2 × 10^3^ cells per chamber) were seeded, and TGF-*β*1 (a final concentration of 0 or 10 ng ml^−1^) and/or Ki26894 (a final concentration of 0, 0.1, 1, or 10 *μ*M) were added to the upper chambers. After 72 h incubation, cells that had not moved to the lower wells were removed from the upper face of the filters using cotton swabs, and the cells that had moved to the lower surface of the filter were stained with haematoxylin. Cancer cells that invaded through a filter coated with matrigel to the lower surface of the membrane were manually counted under a microscope at × 200 magnification. The mean of six fields was calculated as the sample value. The culture was performed in triplicate.

### Immunohistochemistry

BALB/c nude female mice, aged 4 weeks (Nihon CLEA, Tokyo, Japan), were used in the studies. All experiments were performed according to the standard guidelines for animal experiments of Osaka City University Medical School. The expression of T*β*R-I, T*β*R-II, and phosphorylated Smad2 of xenografts was examined. Xenografts were established by injecting each cell (1 × 10^7^) into the flanks of nude mice. Four weeks after inoculation, mice were killed, and the xenografted tumours were washed in PBS and fixed in 10% formalin for paraffin sectioning. Immunohistochemical determination of T*β*R-I, T*β*R-II, and phosphorylated Smad2 was carried out according to the manufacturer’s instructions. In brief, slides were deparaffinised, and were heated for 10 min at 105°C by autoclave in Target Retrieval Solution (Dako, Carpinteria, CA, USA). The sections were then incubated with 3% hydrogen peroxide to block endogenous peroxidase activity. The specimens were incubated with anti-T*β*R-I antibody (Lab vision, Fremont, CA, USA; 1 : 100), anti-T*β*R-II antibody (Lab vision; 1 : 100), and anti-phospho-Smad2 antibody (Chemicon International, Themecula, CA, USA; 1 : 2000) overnight at 4°C. The sections were incubated with biotinylated goat anti-rabbit immunoglobulin G for 30 min. The slides were treated with streptavidin-peroxidase reagent, and were incubated in PBS diaminobenzidine and 1% hydrogen peroxide v/v, followed by counterstaining with Mayer's haematoxylin.

### Western blot analysis

The inhibition by Ki26894 of the phosphorylation of Smad2 and its migration ability in gastric cancer cells were examined by western blotting. Briefly, cell lines were cultured in DMEM with 2% FBS for 2 days. The culture was rinsed with PBS and incubated in serum-free DMEM with reagent (TGF-*β*1: a final concentration of 0 or 10 ng ml^−1^; Ki26894: a final concentration of 0, 0.1, 0.3, 1, 3, or 10 *μ*M) for 60 min. The cells were lysed in a lysis buffer. Aliquots containing 30 *μ*g of total protein were subjected to SDS–PAGE, and the protein bands were transferred to a polyvinylidene difluoride membrane (Amersham, Aylesbury, UK). The membrane was kept in TBS-T (10 mM TBS and 0.05% Tween 20) supplemented with 5% non-fat milk or 5% bovine albumin (Sigma, St Louis, MO, USA) at room temperature for 1 h. Next, the membrane was placed in a TBS-T solution containing each primary antibody. The membrane was placed in TBS-T solution containing the primary antibody, phospho-Smad2 (Cell Signaling Tec, Danvers, CO, USA, Ser 465/467; 1 : 1000), Smad2/3 (Cell Signaling Tec; 1 : 1000), ZO-2 (Cell Signaling Tec; 1 : 1000), phospho-myosin light chain-2 (Cell Signaling Tec, Thr18/Ser19; 1 : 1000), E-cadherin (Cell Signaling Tec; 1 : 1000), or *β*-actin (Cell Signaling Tec; 1 : 1000), and allowed to react at 4°C overnight for western blotting. Next, each antibody was washed three times with TBS-T for 10 min, and a peroxidase-labelled secondary antibody (Amersham) reactive with the primary antibody was added. The membrane was placed in the TBS-T solution, kept at room temperature for 1 h, and then washed. Bands were detected using an enhanced chemiluminescence system (Amersham). An immunoblot analysis was performed twice. PANC-1, a pancreas cancer cell line, was used as the positive control of Smad2 (Romero *et al*, 2008; Horiguchi *et al*, 2009).

### Ras homologue gene family member A activation assay

Activated Ras homologue gene family member A (RhoA) proteins were measured with a RhoA activation G-LISA (Absorbance Based) assay kit (Cytoskeleton, Denver, CO, USA) according to the manufacturer's instructions. The activation of RhoA by TGF-*β*1 and the inhibition by Ki26894 in gastric cancer cells were examined as follows. Cell lines were cultured in DMEM with 2% FBS. The culture was rinsed with PBS and incubated in serum-free DMEM with reagent (TGF-*β*1: a final concentration of 0 or 10 ng ml^−1^; Ki26894: a final concentration of 0 or 10 *μ*M) for 10 min. Activation of RhoA analysis was performed for the third time.

### Statistical analysis

Comparisons among data sets were made with the Kruskal–Wallis one-way ANOVA by ranks, followed by Dunn’s multiple comparison test. A difference was considered significant when the *P*-value was 0.05 or less.

## Results

### Expression of T*β*R-I, T*β*R-II, and phospho-Smad2 in gastric cancer cells

**Figure 1:**
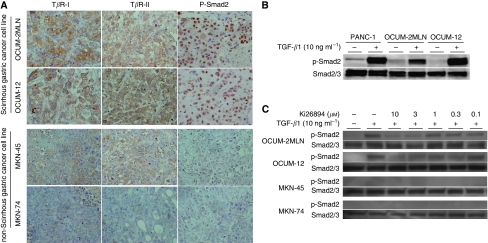
Expression of TGF-*β* signalling and inhibitory effect of Ki26894. (**A**) Expression level of TGF-*β* receptor and phospho-Smad2. The overexpression of TGF-*β* receptor type I (T*β*R-I), type II (T*β*R-II), and phospho-Smad2 (P-Smad2) was observed in scirrhous gastric cancer cell lines, OCUM-2MLN and OCUM-12, but not in non-scirrhous gastric cancer cell lines, MKN-45 and MKN-74. (**B**, **C**) Effects of Ki26894 on TGF-*β-*induced Smad2 phosphorylation of gastric cancer cells. PANC-1 was used as positive control of Smad2 phosphorylation. Transforming growth factor-*β* stimulated Smad2 phosphorylation in PANC-1, OCUM-2MLN, and OCUM-12 cells. Total Smad2/3 expression was recognised in all four gastric cancer cell lines and no difference in expression level was found on addition of TGF-*β*1 or Ki26894. Transforming growth factor-*β*1 (10 ng ml^−1^) stimulated Smad2 phosphorylation in both scirrhous gastric cancer cell lines, but not in non-scirrhous cancer cell lines, MKN-45 and MKN-74. Smad2 phosphorylation was decreased in a dose-dependent manner by Ki26894 in scirrhous gastric cancer cell lines. PANC-1, a pancreas cancer cell line, was used as positive control of Smad2.

Figure 1A shows the expression level of T*β*R-I, T*β*R-II, and phospho-Smad2 of gastric cancer cells in xenografted tumours. T*β*R-I and T*β*R-II were immunolocalised at the membrane and cytoplasm, respectively, of gastric cancer cells. High expression levels of T*β*R-I and T*β*R-II were found in scirrhous gastric cancer cells (OCUM-2MLN and OCUM-12), but not in non-scirrhous gastric cancer cells (MKN-45 and MKN-74). Phospho-Smad2 was expressed in the nucleus of scirrhous gastric cancer cells. The overexpression of phospho-Smad2 was observed in scirrhous gastric cancer cells, but not in non-scirrhous gastric cancer cells.

### Effects of Ki26894 on Smad2 phosphorylation in gastric cancer cells

To determine whether the small-molecule compound, Ki26894, inhibits TGF-*β* signalling, the effect of Ki26894 on TGF-*β*-induced Smad2 phosphorylation was examined in gastric cancer cells. Smad2 phosphorylation was increased by TGF-*β*1 (10 ng ml^−1^) in scirrhous gastric cancer cell lines, OCUM-2MLN and OCUM-12. Smad2 phosphorylation was decreased in a dose-dependent manner by Ki26894 from 3 to 10 *μ*M. In contrast, Smad2 phosphorylation was not detected with TGF-*β*1 treatment in non-scirrhous gastric cancer cell lines, MKN-45 and MKN-74 (Figure 1B, 1C).

### Ki26894 reduces the migration of scirrhous gastric cancer cells induced by TGF-*β*

**Figure 2:**
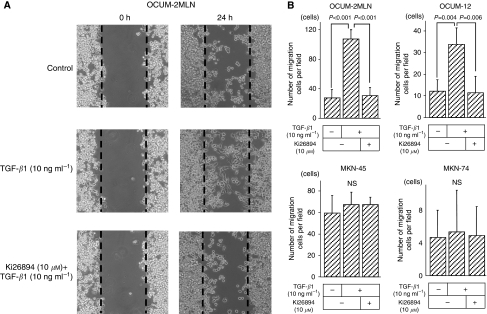
Effect of Ki26894 on the wound healing ability of cancer cells. (**A**) Representative pictures from three experiments are shown. The number of OCUM-2MLN cells that migrated over the wound line (dotted line) was increased by TGF-*β*1 treatment when compared with that of control. The migrating cells displayed spindle shapes characteristic of epithelial-to-mesenchymal transition (EMT). Ki26894 (10 *μ*M) downregulated the ability of OCUM-2MLN cells to migrate over a wound line. (**B**) TGF-*β*1 significantly stimulated the migration of scirrhous gastric cancer cells, but not of non-scirrhous gastric cancer cells. Ki26894 (10 *μ*M) significantly inhibited the migration of scirrhous gastric cancer cells.

Figure 2A is a representative phase-contrast photograph of OCUM-2MLN cells. The number of migrating OCUM-2MLN cells was increased by TGF-*β*1 (10 ng ml^−1^) and was decreased in the presence of Ki26894 (10 *μ*M). The migrating ability of OCUM-12 cells was increased in the presence of TGF-*β*1 (Supplementary Movie 1), compared with the control (Supplementary Movie 2). Transforming growth factor-*β*1 significantly stimulated the migration of OCUM-2MLN (*P*<0.001) and OCUM-12 cells (*P*=0.004). Ki26894 significantly inhibited the migration-stimulating ability of TGF-*β*1 in both OCUM-2MLN (*P*<0.001) and OCUM-12 cells (*P*=0.006). In contrast, neither TGF*β*1 nor Ki26894 affected migration by the non-scirrhous gastric cancer cell lines used (Figure 2B).

### Effect of Ki26894 on scirrhous gastric cancer cell invasion

**Figure 3:**
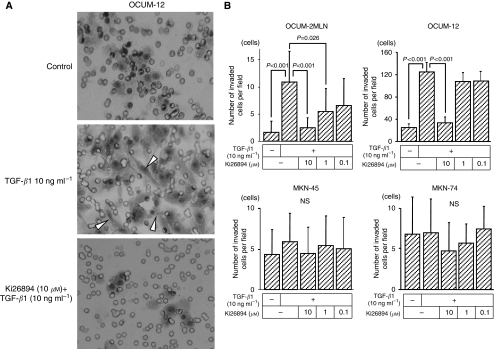
Ki26894 inhibits invasion by scirrhous gastric cancer cells. (**A**) Representative pictures of invading gastric cancer cells. OCUM-12 cells invaded into a 12 *μ*m-pore membrane filter in the presence of TGF-*β*1 when compared with control. Most of the invaded cells show epithelial-to-mesenchymal transition (arrow heads). Ki26894 (10 *μ*M) inhibited the invasion induced by TGF-*β*1. (**B**) Transforming growth factor-*β*1 significantly stimulated the invasive behaviour of OCUM-2MLN and OCUM-12 cells. Ki26894 (10 *μ*M) significantly inhibited the invasion seen in these cells. In contrast, invasion by non-scirrhous gastric cancer cell lines, MKN-45 and MKN-74, was not affected by TGF-*β*1 or by Ki26894.

Figure 3A is a representative phase-contrast photograph of OCUM-12 cells that have invaded into a 12-*μ*m-pore membrane filter. The number of OCUM-12 cells displaying EMT was significantly increased in the presence of TGF-*β*1 when compared with the control. The migration-stimulating activity of TGF-*β*1 was decreased in the presence of Ki26894 (10 *μ*M). Transforming growth factor-*β*1 (10 ng ml^−1^) significantly stimulated invasion (*P*<0.001) by scirrhous gastric cancer cell lines, but not by non-scirrhous gastric cancer cell lines. Ki26894 (10 *μ*M) significantly (*P*<0.001) inhibited invasion by scirrhous gastric cancer cells. In contrast, invasion was not affected by TGF-*β*1 or Ki26894 in the non-scirrhous gastric cancer cell lines used (Figure 3B).

### Effects of TGF-*β*1 and Ki26894 on the morphological characteristics of gastric cancer cells

**Figure 4:**
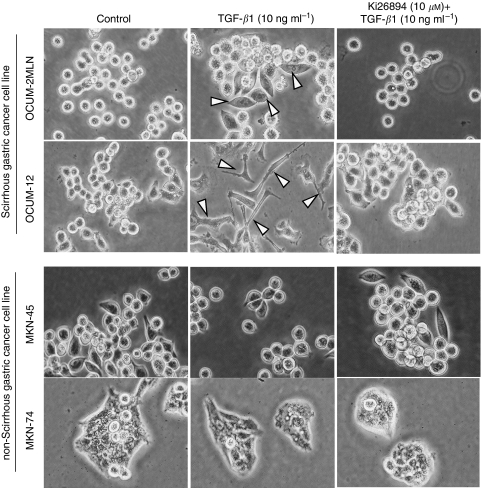
Effects of TGF-*β*1 and Ki26894 on morphological characteristics of gastric cancer cells. Epithelial-to-mesenchymal transition (arrow heads) by TGF-*β*1 (10 ng ml^−1^) was shown in scirrhous gastric cancer cell lines, OCUM-2MLN and OCUM-12, but not in non-scirrhous gastric cancer cell lines, MKN-45 and MKN-74. Pseudopod formation was remarkable in OCUM-12 cells in the presence of TGF-*β*1. Epithelial-to-mesenchymal transition by TGF-*β*1 in scirrhous gastric cancer cells was inhibited by Ki26894 (10 *μ*M).

Epithelial-to-mesenchymal transition was found in both scirrhous gastric cancer cell lines (OCUM-12 and OCUM-2MLN) in culture with the addition of TGF-*β*1. An increase in the number of attached and spreading cells was found after the addition of TGF-*β*1, whereas most of the OCUM-2MLN and OCUM-12 cells without TGF-*β*1 treatment were still round. OCUM-12 cells exhibited loss of cell–cell adhesion and spindle-shaped cells. However, Ki26894 (10 *μ*M) inhibited these morphological changes associated with EMT in scirrhous gastric cancer cells. In contrast, non-scirrhous gastric cancer cell lines (MKN-45 and MKN-74) did not display these morphological changes after the addition of TGF-*β*1 (Figure 4).

### Effects of Ki26894 on cellular signalling pathways exhibited during migration

**Figure 5:**
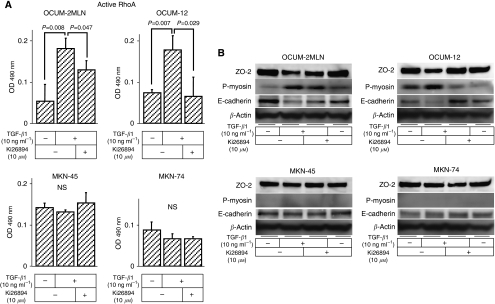
Effects of Ki26894 on cellular migration signals in gastric cancer cells. (**A**) ELISA of RhoA. Transforming growth factor-*β*1 (10 ng ml^−1^) significantly upregulated the active form of RhoA in scirrhous gastric cancer cell lines, OCUM-2MLN and OCUM-12, and Ki26894 (10 *μ*M) significantly inhibited this effect. In contrast, the active form of RhoA was not increased by TGF-*β*1 or by Ki26894 in non-scirrhous cancer cell lines, MKN-45 and MKN-74. (**B**) Transforming growth factor-*β*1 (10 ng ml^−1^) increased myosin light chain-2 phosphorylation (p-myosin) and decreased ZO-2 and E-cadherin expression in scirrhous gastric cancer cell lines, but not in non-scirrhous gastric cancer cell lines. Ki26894 (10 *μ*M) decreased p-myosin, and increased ZO-2 and E-cadherin expression in scirrhous gastric cancer cell lines.

Transforming growth factor-*β*1 significantly upregulated the active form of RhoA in scirrhous gastric cancer cell lines, OCUM-2MLN (*P*=0.008) and OCUM-12 (*P*=0.007), when compared with control. However, Ki26894 significantly inhibited the active RhoA induced by TGF-*β*1 in scirrhous gastric cancer cell lines, OCUM-2MLN (*P*=0.047) and OCUM-12 (*P*=0.029). In contrast, active RhoA was not affected by TGF-*β*1 or Ki26894 in non-scirrhous cancer cell lines (Figure 5A). Transforming growth factor-*β*1 (10 ng ml^−1^) decreased ZO-2 and E-cadherin expression in scirrhous gastric cancer cells and increased myosin light chain-2 phosphorylation (p-myosin), but not in non-scirrhous gastric cancer cells. In addition, Ki26894 increased ZO-2 and E-cadherin expression and inhibited p-myosin in scirrhous gastric cancer cells (Figure 5B).

## Discussion

In this study, scirrhous gastric cancer cells expressed T*β*R-I and T*β*R-II. Furthermore, TGF-*β* stimulated the phosphorylation of Smad2 in scirrhous gastric cancer cells as previously reported (Komuro *et al*, 2009), but not in non-scirrhous gastric cancer cells, suggesting that TGF-*β* signalling is active in scirrhous gastric cancer, but not in non-scirrhous gastric cancer.

Transforming growth factor-*β*1 stimulated EMT in scirrhous gastric cancer cells, but not in non-scirrhous gastric cancer cells. Epithelial-to-mesenchymal transition is characterised by spindle-shaped cells with a reduction in epithelial cell markers of cell–cell adhesion molecules (Thiery, 2002; Jung *et al*, 2006; Revenu and Gilmour, 2009). The expression levels of cell–cell adhesion molecules, ZO-2 and E-cadherin, were downregulated by TGF-*β*1 in scirrhous gastric cancer cells. Moreover, T*β*R signalling activates RhoA, which mediates the upregulation of myosin light chain-2 phosphorylation (Shook and Keller, 2003; Fan *et al*, 2007) and downregulation of cell–cell tight junctions (Takaishi *et al*, 1997; Yamazaki *et al*, 2008). Transforming growth factor-*β*1 significantly upregulated active RhoA and phospho-myosin of scirrhous gastric cancer cells. The EMT of scirrhous gastric cancer cells induced by TGF*β* signalling might be mediated by the regulation of RhoA, myosin, ZO-2, and E-cadherin. Scirrhous gastric carcinoma is a diffuse-type gastric cancer characterised by loss of cell–cell adhesion. Transforming growth factor-*β* signalling of downregulation of cell–cell adhesion might be closely associated with the histologenesis of scirrhous gastric carcinoma. Intestinal-type tumours show an expanding growth with definite border according to the Laurén classification (Lauren, 1965). These histological differences between diffuse-type and intestinal-type tumours might be determined in part by the response of gastric cancer cells to TGF*β*/T*β*R signalling.

Transforming growth factor-*β*R signalling increased the migrating ability of scirrhous gastric cancer cells, but not of non-scirrhous gastric cancer cells. Transforming growth factor-*β*1 activates RhoA, which promotes cancer cell invasion and eventually leads to metastasis by disrupting E-cadherin-mediated adherens junctions (Chang *et al*, 2009). In this study, TGF-*β*1 significantly upregulated active RhoA of scirrhous gastric cancer cells. Ras homologue gene family member A might regulate not only EMT but also migrating ability in scirrhous gastric cancer cells through TGF*β* signalling. High migration ability of scirrhous-type cancer cells by TGF*β* signalling is one of the reasons responsible for a diffusely infiltrating growth with an indistinct border from the surrounding tissue. Scirrhous carcinomas carry a worse prognosis than other types of gastric carcinomas, reflecting a high frequency of metastasis (Otsuji *et al*, 2004; Ikeguchi *et al*, 2009). Previous studies have reported that the phenotypes of metastatic cancer cells are associated with migration ability (Jung *et al*, 2006; Revenu and Gilmour, 2009). The different response in TGF-*β*/T*β*R signalling between scirrhous types and non-scirrhous types might explain the poorer prognosis of scirrhous-type gastric cancers compared with non-scirrhous-types of gastric cancers.

Transforming growth factor-*β* is produced not only by scirrhous gastric cancer cells (Yoshida *et al*, 1989; Niki *et al*, 2000) but also by cancer-associated fibroblasts (Mizoi *et al*, 1993; Inoue *et al*, 1997; Zeisberg *et al*, 2007). Our preliminary study recognised that both scirrhous gastric cancer cell lines and gastric fibroblasts produced TGF-*β* (Yashiro *et al*, 1996b; Inoue *et al*, 1997). These findings suggest that the invasive capacity induced by TGF-*β* may be influenced in both an autocrine and paracrine manner. Tumour cells in scirrhous carcinoma produce more TGF*β*, which is a key mediator of fibroblast activation, than those in non-scirrhous carcinoma (Yoshida *et al*, 1989; Mahara *et al*, 1994). Scirrhous gastric carcinoma is characterised by cancer cell infiltration accompanied by extensive stromal fibrosis (Yashiro *et al*, 1996a). The typical histological findings of rapid infiltration with fibrosis might indicate that the surrounding stromal fibroblasts contribute to cancer progression more intensely in scirrhous gastric cancer than in non-scirrhous gastric cancer.

Specific small molecules designed to inhibit the effects of TGF-*β* at the level of signalling receptors may be a promising strategy for patients with advanced gastric carcinoma. In this study, a T*β*R-I inhibitor, Ki26894, displayed inhibitory activity against phosphorylation of Smad2 in scirrhous gastric cancer, suggesting that the small-molecule compound Ki26894 is a potent T*β*R kinase inhibitor. Migration and invasion stimulated by TGF-*β* were significantly inhibited by Ki26894 in scirrhous gastric cancer cells. In contrast, the migration and invasion by non-scirrhous cancer cells were not affected by TGF-*β*1 or by Ki26894. Cell motility and invasion are critical metastatic events in cancer progression (Ehata *et al*, 2007). Ki26894, a small molecule designed to inhibit T*β*R, might be a promising therapeutic agent for antagonising the metastatic phenotypes seen through autocrine TGF-*β*/T*β*R signalling in scirrhous gastric cancer. We previously reported that the T*β*R inhibitor, A-77, decreased the expression of integrins in cancer cells, which resulted in a decreased adhesion of scirrhous gastric cancer cells to peritoneum (Kawajiri *et al*, 2008). Furthermore, Ki26894 inhibited the upregulation of active RhoA and phospho-myosin and inhibited the downregulation of E-cadherin and ZO-2 expression in scirrhous gastric cancer cells. Invasion by scirrhous gastric cancer cells may be inhibited by a T*β*R inhibitor through regulation of RhoA, myosin, E-cadherin, and ZO-2 expression. These findings suggested that inhibition of the biological effects of TGF-*β* might affect the adhesion, migration, and invasion of cancer cells and may be an attractive strategy for prevention of distant metastasis by scirrhous gastric cancers.

In conclusion, TGF*β* signalling stimulated the EMT and invasion ability of scirrhous gastric cancer cells through regulation of RhoA, myosin, ZO-2, and E-cadherin. Ki26894, which inhibits T*β*R-I phosphorylation, significantly inhibited invasion by scirrhous gastric cancer cells. The T*β*R might be a promising target molecule for the treatment of scirrhous gastric carcinoma.
