# Supplementary material for: Inhibitory effect of a TGFβ receptor type-I inhibitor, Ki26894, on invasiveness of scirrhous gastric cancer cells
Source: Br J Cancer. 2010 Feb 9;102(5):844–51. doi: 10.1038/sj.bjc.6605561 (PMC2833252; doi:10.1038/sj.bjc.6605561)
Supplement: Supplementary Movie Legends [file 6605561x3.doc]

**Supplement movie.** Real-time living-cell imaging of OCUM-12 cells was monitored by time-lapse video microscopy for 72 h at 15-min intervals. An increase in the number of spindle-shaped and spreading OCUM-12 cells was found following the addition of TGF-β1 **(Supplementary movie 1)**, while the control OCUM-12 cells displayed round shapes and low migration **(Supplementary movie 2)**.
